# Exploring the spiritual experiences of older adults with chronic diseases: a qualitative study in a multicultural context

**DOI:** 10.1186/s40359-025-02710-3

**Published:** 2025-07-01

**Authors:** Linan Cheng, Yan Liu, Qian Chen, Fengying Zhang

**Affiliations:** 1 School of Nursing, Wenzhou Medical University, Wenzhou, Zhejiang China; 2 West China School of Public Health and West China Fourth Hospital, West China Nursing School, Sichuan University, Chengdu, Sichuan China; 3 West China School of Nursing/West China Hospital, Sichuan University, Chengdu, Sichuan China

**Keywords:** Older adults, Chronic disease, Spirituality, Spiritual needs, Spiritual experience

## Abstract

**Background:**

Meeting spiritual needs of older adults with chronic illnesses in multicultural settings is challenging but vital for well-being, respect, and global health equity.

**Methods:**

We conducted a qualitative study in culturally diverse regions of China to explore the spiritual experiences of older adults living with chronic diseases. Semi-structured, face-to-face interviews were conducted with a total of 28 participants, comprising 25 individuals suffering from chronic illnesses and 3 healthcare professionals. An inductive content analysis approach was utilized to analyze the data.

**Results:**

Seven themes emerged, encompassing spiritual awareness, spiritual needs, spiritual resources, spiritual growth, spiritual equilibrium, spiritual outreach, and spiritual cultivation. The proposed model illustrates a spiraling growth trend in the spiritual experience of older adults. Regardless of their awareness of spirituality, the aging process prompts individuals to explore their spiritual needs and engage in spiritual experiences to varying extents.

**Conclusions:**

Addressing spiritual needs of older adults with chronic illnesses in multicultural context is vital. Respecting cultural diversity and fostering spiritual growth enhances dignity, autonomy equity, and well being of older adults. The study model reveals dynamic spiritual experiences, highlighting the need for individualized interventions.

**Clinical trial number:**

Not applicable.

**Supplementary Information:**

The online version contains supplementary material available at 10.1186/s40359-025-02710-3.

## Introduction

The global population is undergoing a rapid process of aging, adding to the steady rise in the proportion of senior citizens (aged over 65) [1]. A study shows that 180 million Chinese older adults suffered from chronic diseases in 2019, accounting for 75.8% of the total, with projections indicating a surge in the coming decades [2]. Spirituality has emerged as a vital asset in active aging, intricately woven into the fabric of coping with the aging process and caring for older adults [3–4]. Consequently, addressing spirituality in the medical and mental healthcare of older adults globally, particularly those afflicted with chronic illnesses, is garnering growing attention within the scholarly literature [5].

“Spirituality is a dynamic and intrinsic aspect of humanity through which persons seek ultimate meaning, purpose, and transcendence, and experience relationships with themselves, their family members, communities, society, and nature with the significant or sacred. Spirituality is expressed through beliefs, values, traditions, and practices” [6]. Therefore, spirituality can be defined as the pursuit of meaning, purpose, connection, and transcendence. Given the rich life experiences of older adults, spirituality is often viewed as a coping mechanism or a form of social support intervention in the face of illnesses and crisis events [7]. Moreover, spirituality serves as a source of strength, providing a framework for comprehending illness, death, and the mysteries of life. It fosters a sense of hope, resilience, and even contributes to improved disease management and an enhanced overall quality of life [8]. Especially for older adults with chronic illnesses, understanding how spirituality intersects with these health challenges is vital for developing comprehensive and holistic care strategies [9].

It is noteworthy that spirituality is inherently characterized by cultural sensitivity, as cultural sensitivity acknowledges that each individual’s spiritual experience is deeply intertwined with their unique cultural values [10]. Consequently, the exploration of spirituality within healthcare necessitates adherence to ethical principles that safeguard the dignity, autonomy, and privacy of patients. Medical ethics dictate that healthcare professionals must uphold a steadfast commitment to respecting patients’ spiritual beliefs and practices, even when they differ from their own [11]. The ethical principles of cultural sensitivity necessitate that healthcare professionals maintain a stance of cultural openness and inclusivity, which is extremely important within a multicultural society [12]. This approach recognizes the profound interconnectedness between an individual’s spiritual experiences and their cultural heritage. This ethical obligation encompasses refraining from imposing personal views and abstaining from any discriminatory practices that stem from cultural or religious differences. By adhering to these ethical principles, we can ensure that patients feel safe and empowered to share their spiritual experiences, which can serve as a catalyst in their healing journey.

To harness the role of spirituality in addressing the health challenges of older adults with chronic disease, accurately identifying their spiritual needs is a crucial step in providing appropriate spiritual care interventions [13]. Studies have reported that older adults with chronic disease have strong spiritual needs [13–14]. Met spiritual needs can bring confidence and hope [15], help individuals overcome life’s challenges [16–17], and cope with loss and change in later life, especially in terms of decreased physical health function and dependence [18–19]. Conversely, if spiritual needs are not met, individuals cannot reach their optimal health potential [20]. Thus, the spiritual needs of older adults with chronic disease deserve greater attention.Qualitative research provides unparalleled insights into spiritual needs in chronic illness that quantitative methods cannot achieve. This approach uniquely explores how spirituality is subjectively constructed and dynamically adapted throughout disease progression [4]. It particularly excels in revealing culturally contextualized spiritual expressions and the interpersonal dynamics influencing spiritual adaptation within caregiving relationships.Moreover, qualitative methodology offers distinctive access to existential dimensions of chronic illness, including meaning-making processes and spiritual distress that elude quantitative measurement. Such in-depth understanding is essential for developing interventions that genuinely address patients’ and caregivers’ spiritual needs within their sociocultural contexts. This study employs qualitative inquiry to investigate these complex phenomena, contributing to the development of culturally sensitive spiritual care practices.

It is imperative that cultural sensitivity be more prominently addressed in multicultural countries.China is a multiethnic country with a long cultural tradition. Traditional Chinese culture is the general term used to refer to Chinese national civilization, customs and spirituality, with Confucianism, Buddhism and Taoism as the main belief systems. These belief systems depend on each other, penetrate each other and influence each other to build the whole of traditional Chinese culture [21].

In Chinese culture, family, kinship, and social relationships are central to shaping an individual’s spirituality and sense of life meaning. Values such as “family loyalty,” respect for elders, and a family-centered social structure profoundly influence the spiritual experiences and needs of older adults. For those with chronic diseases, spiritual needs are closely linked to family dynamics, social responsibilities, and cultural values. These cultural factors may lead to distinct expressions and understandings of spiritual growth compared to Western cultural contexts. Therefore, this study focuses on older adults within the Chinese cultural framework, reflecting the cultural context of Chinese society, while recognizing the diversity of spiritual needs and experiences across different cultures.

Multiculturalism, as it occurs, enriches our comprehension of spirituality by acknowledging the diversity of beliefs, practices, and values across various ethnic, racial, and religious groups [22]. Moreover, the spiritual needs of older adults in the Chinese cultural context are still in the exploratory stage. One of the prominent phenomena of “spiritual care” in China is the gap between the extreme desire for spiritual resources and the extreme lack of spiritual resources [23]. And the spiritual experiences of older adults with chronic illnesses are closely interconnected with those of their caregivers. Caregivers’ spirituality not only plays a key role in influencing the well-being of patients but also shapes the quality of care provided. Furthermore, caregivers’ spiritual understanding is essential in identifying and addressing the spiritual needs of patients [21]. Therefore, incorporating caregivers’ perspectives is crucial for a comprehensive understanding of the spiritual needs of older adults with chronic diseases.

In this context, it is essential to explore the spiritual experience of older adults with chronic disease in the Chinese context.Through this study, we aim to foster the exploration of spirituality research within a multicultural context, enabling participants to comfortably share their spiritual experiences without judgment or discrimination. This ensures the safeguarding of participants’ rights, dignity, and privacy, thereby promoting spiritual well-being and global health equity.

## Methods

A qualitative descriptive study design was employed. Data were collected through semi-structured face-to-face interviews and structured assessment tools. The study’s reporting followed the Consolidated Criteria for Reporting Qualitative Studies (COREQ) [24], along with guidance from Whiting (2008) on semi-structured interviews [25].

### Setting

Given the vast cultural, geographical, and socioeconomic disparities in China, participants were recruited from northern China (two hospitals), central China (a community hospital), and southern China (one hospital). The hospitals in the north and south were large tertiary hospitals with established referral relationships with the local community. Notably, one of the northern hospitals had a significant Korean ethnic minority patient population. Due to the COVID-19 pandemic, older adults from the community health service center were interviewed via online video.

Inclusion Criteria: (1) age ≥ 60 years old; (2) diagnosis of at least one chronic illness confirmed by a physician from a tertiary hospital; and (3) provision of informed consent by themselves and their families. Exclusion criteria: (1) inability to complete the whole interview process; (2) the initial interview lasting less than 20 min. and (3) interruption of the video-based interview for any reasons. All samples where a clear and effective interview could not be obtained were excluded from the data analysis.

To gain a comprehensive understanding of spirituality, the study also encompassed health caregivers who provide care for older adults. The inclusion criteria for these caregivers were as follows: (1) having more than five years of experience caring for older adults with chronic diseases; (2) demonstrating a concern or interest in spiritual studies; and (3) providing informed consent to participate. The exclusion criterion was failure to complete the interview for any reason.

### Sample size

The sample size was determined by thematic saturation criteria [1], operationalized through three established indicators: (a) absence of new substantive codes in three consecutive interviews, (b) comprehensive development of all identified thematic categories, and (c) confirmation through negative case analysis [26].

### Data collection

The data were collected between July 2020 and June 2022. Before data collection, the researchers developed a semi-structured interview guide based on the literature, expert advice, and input from potential interviewees. Additionally, an observer took field notes. The study followed the subtle realist paradigm [25], according to which reality is conceptualized as existing objectively [27], but known only from each individual’s own perspective [6]. Data collection was informed by the concept of spirituality as well as traditional Chinese culture and sought to understand the perspectives of older adults and caregivers. We defined a series of questions focusing on understanding spirituality, spiritual needs and the need for spiritual care, but participants were encouraged to express their opinions as much as possible (Appendix 1). Meanwhile, we asked in-depth questions based on their answers (2 participants repeated the interviews in the study by the video network).

After each interview, the researcher summarized the content proposed and made improvements in the next interview according to whether new topics appeared in the interview content. Data collection was concluded when no new topics emerged and saturation was reached.

### Trustworthiness

To ensure methodological rigor, we implemented a comprehensive quality assurance protocol. Five pilot interviews were initially conducted to culturally adapt and linguistically optimize the interview guide for geriatric populations while preserving conceptual precision. Participants received the finalized interview guides 24 h in advance to facilitate adequate preparation and reflection. All interview sessions, lasting 35–70 min, were conducted in a controlled environment (soundproof conference room) during participants’ optimal time windows (post-treatment or rest periods). Audio-visual recordings were obtained following written informed consent procedures, with ongoing verbal confirmation of participant comfort throughout the sessions. These measures collectively enhanced study credibility through prolonged engagement and iterative member validation.

The research team employed systematic triangulation strategies to ensure confirmability. A qualified female interviewer (doctoral candidate in palliative care with three years of geriatric clinical experience and two years of dedicated qualitative research practice) conducted all interviews under the supervision of two senior qualitative methodologists. Regular peer debriefing sessions were conducted following each interview to minimize researcher bias. We maintained a comprehensive audit trail documenting all procedural adaptations, coding rationales, and analytical decisions. Intercoder reliability was rigorously established (κ = 0.81, 95% CI 0.76–0.85) through dual independent analysis of a randomly selected 20% sample of transcripts, supplemented by structured member checking with five participants to verify interpretive accuracy.

To ensure transferability, we collected detailed contextual data including: (1) comprehensive participant demographics (age, education, religious belief), (2) environmental particulars (clinical setting characteristics), and (3) researcher positionality statements (documenting the interviewer’s professional background and ongoing mentorship by palliative care specialists).The study protocol received formal ethical approval from the Institutional Review Board of West China Hospital, Sichuan University (Ethics Approval No.2020 − 697).

### Analysis

The interview contents were transcribed verbatim within 24 h after the interview, and the text arrangement was completed one week after the interview. We used the inductive content analysis method to analyse data; this method is appropriate when the phenomenon is not well understood and relevant theoretical guidance is lacking [28]. The data analysis employed a systematic three-phase inductive approach: (1) Preparation phase, comprising iterative transcript review and identification of meaning units; (2) Organization phase, utilizing Nvivo 12 (QSR International) for open coding with concurrent codebook refinement; and (3) Abstraction phase, involving category development through constant comparative analysis and thematic validation via member checking and peer debriefing procedures.

Two researchers (LH and LNC) independently developed the coding frames and wrote notes and headings in the margins of the document. Then, they discussed, compared and coded the data until they reached an agreement. After coding, categories were generated based on high-level headings [29]. Two researchers sorted the data. In case of disagreement, an experienced researcher (QC) participated in discussion until they reached an agreement. The last step was to extract the data and determine the themes [30]. Sub-themes were grouped to determine the themes, data were extracted from direct citations, and a report was created. In this study report, M and F (for male and female) refer to the study participants, and N refers to their caregivers, respectively.

## Results

### Participant characteristics

Through purposive sampling, we engaged with 28 participants (25 older adults with chronic conditions and 3 health caregivers) who provided rich narratives about their spiritual experiences. The included participants represented diverse demographic and clinical characteristics (see Table 1).

Three female geriatric caregivers (N1, N2, N3) were recruited for the Geriatric Medical Center. All married and aged 38–50, N1 holds a Ph.D. and serves as Chief Nurse/Professor, N2 is a devout Buddhist with an M.S. and acts as Supervisor Nurse, while N3, also a Supervisor Nurse, holds a B.S. Together, they contribute to geriatric care services.

**Table 1:** Demographic characteristics of participants (*n* = 25)

Characteristics	Modalities	*n* (%) or Mean ± SD
Age (years)		70.6 ± 6.47
	≤ 70	14 (56)
	71 ~ 82	11 (44)
Gender	Women	19 (76)
	Men	6 (24)
Marital status	Divorced	1 (4)
	Widowed	4 (16)
	Married	20 (80)
Education	Primary school	5 (20)
	Junior high school	3 (12)
	Senior high school	11 (44)
	Undergraduate	4 (16)
	Technical secondary school	2 (8)
Religious belief	No	15 (62.5)
	Buddhism	4 (16.67)
	Christianity	4 (16.67)
	Folk belief	1 (4.17)
Number of Chronic Diseases	1	15 (60)
	≥ 2	10 (40)
Interview time		44.52 ± 9.59
	≤ 45	15(60)
	46 ~ 65	10(40)

### The theoretical model of the spiritual experience

**Fig. 1:**
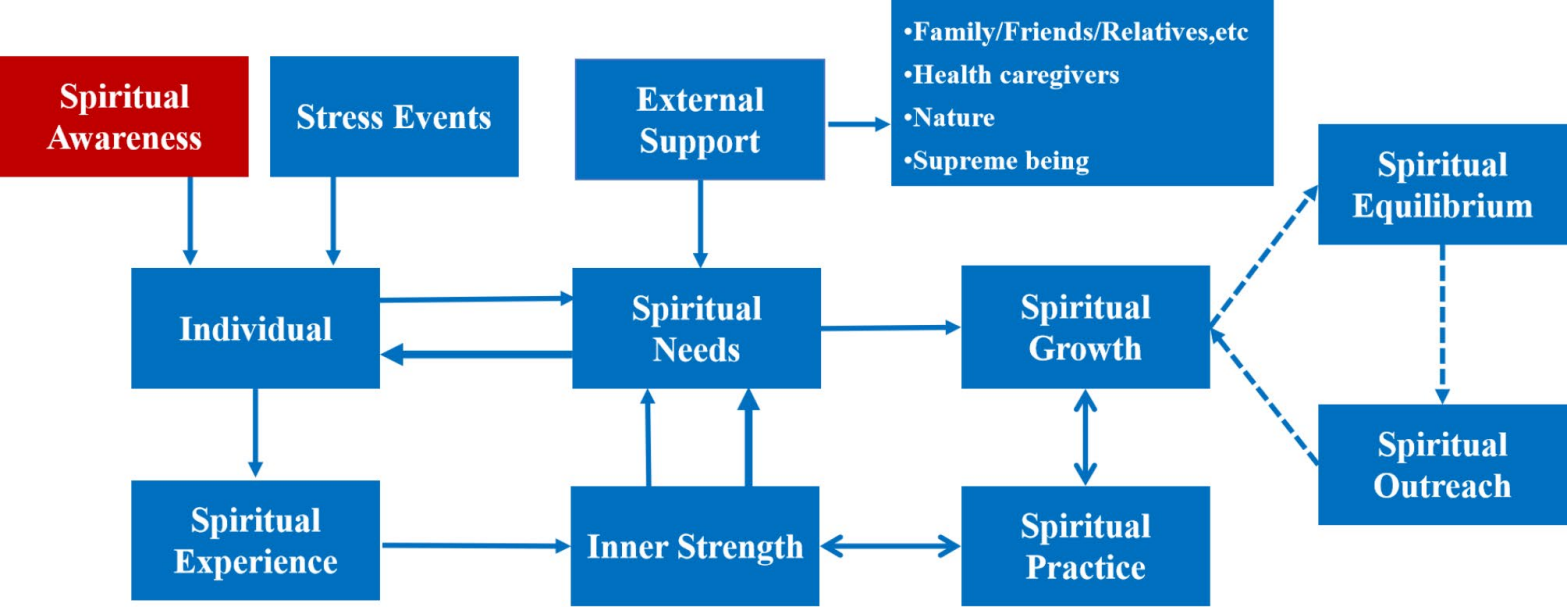
The theoretical model of the spiritual experience of older adults with chronic diseases

A total of seven themes have emerged from analysis. These are spiritual awareness, spiritual needs, spiritual resources, spiritual growth, spiritual equilibrium, spiritual outreach, and spiritual practice. And the theoretical model of the spiritual experience of older adults was explored. Regardless of whether older adults know about spirituality, ageing can lead individuals to explore their spiritual needs and experience spirituality to some extent. Spiritual needs promote individual spiritual growth through external support and/or inherent strength under different circumstances or stressful events. After experiencing spiritual strength, spiritually developed individuals master spiritual capacity, which promote these individuals to maintain their spirituality. Some individuals promote spiritual outreach by helping others. This expanded spirituality further increases individuals’ spiritual growth and thus enhances their inner strength. Additionally, there exists a bidirectional, mutually reinforcing relationship between individual spiritual practice and both spiritual growth and internal strength, creating a cyclical process that ultimately enhances individuals’ spiritual experiences and awareness. However, due to the lack of spiritual research in China, some individuals have spiritual experience but are not aware of it (see Fig. 1).

### Spiritual awareness

The older adults interviewed in this study rarely grasped the concept of spirituality, yet all of them had experienced spirituality in some form. The healthcare providers, on the other hand, possessed a certain level of understanding of spirituality, albeit not explicitly defined, and demonstrated a degree of discernment in this realm. Spiritual awareness, in this context, pertains to the perception of spiritual existence, the existential dimension, spiritual capacity, a sense of spiritual transcendence, and the dynamic principle governing spiritual transformation.

The interviewer mentioned that spirituality is intangible and requires perception. Non-religious individuals find it challenging to define spirituality. While spirituality exists, it often goes unnoticed in the subconscious. This doesn’t mean it doesn’t exist, but rather it hasn’t been thoroughly explored. Spirituality can sometimes aid in problem-solving and be expressed as joy or contentment. For religious believers, spirituality may manifest as a belief in a guiding force, akin to a sixth sense or momentary intuition.

*‘People possess diverse mindsets. Being content can bring us tranquility and gratitude. Perhaps this mindset embodies the spirituality you mentioned’(M2)*.

*‘I believe in Jesus. He gives me strength*,* peace*,* and courage. It’s like breathing; when we’re born*,* it’s as if Jesus breathes life into us*,* and we have a spirit. And when we die*,* that last breath signals the soul’s departure.’(F12)*.

*‘Spirituality includes all human senses. When I explore within*,* I feel a divine presence. Sometimes*,* I feel very spiritual.’(F13)*.

*‘It (Spirituality) may seem elusive*,* but it undeniably exists. Defining or conceptualizing its existence proves challenging. It possesses an air of mystery*,* yet it appears to be real*,* as it sometimes offers solutions to specific issues.’ (F16)*.

Spirituality can be understood as a construct shaped by the interplay of time, place, and individuals. Within this framework, energy is continuously transformed. Under the excitation of the environment, energy is transformed in three-dimensional space. For older adults, the profound impression of a particular aspect from the past transforms into their spiritual experience, and now, through memory, it evolves into a source of spiritual strength.

*‘As people age*,* they often find solace in reminiscing about someone*,* something*,* or a place from the past. I believe this represents a spiritual connection to the three dimensions of the past-time*,* place*,* and people.’ (N2)*.

Spiritual individuals can live in peace and contentment in any environment. Spirituality is a strength, more like an ability, that helps older people through difficulties in all stages of life, and spirituality can also provide the strength and courage to overcome suffering through religious faith.

*‘Even during times of suffering chronic diseases*,* I turn to prayer*,* and I am unafraid when I have the Spirit of God within me.’ (F10)*.

*‘As I near 80 years of age and face countless challenges*,* including gradually worsening chronic diseases*,* I do not feel weary; my faith gives me strength.’ (F13)*.

*‘Spirituality can help me inwardly accept chronic illness with calmness.’(F18)*.

Transcendence is manifested in spirituality, which encompasses religion but stands apart from it. Spirituality transcends mere physical and mental constructs, and it represents a form of contentment that surpasses material desires. This spiritual contentment aids elderly individuals with chronic illnesses in transcending the hardships brought about by physical aging, sudden stressful events, and the relentless struggle with disease.

*‘Spirituality*,* which transcends the boundaries of religion*,* has the power to alleviate the physical suffering caused by illness.” (F16)*.

*‘I firmly believe that spirituality enables many elderly individuals with chronic illnesses to transcend both physical and mental realms*,* encompassing profound aspects such as the meaning of life and self-awareness.’(N1)*.

*‘While psychological needs may seem superficial due to their observability*,* the needs originating from the depths of one’s spirit are distinctly different and profound.” (F20)*.

*‘Spirituality is about being content with what one has. In old age*,* facing illnesses and the inevitable separation from loved ones is a natural part of life that must be embraced. Despite my current struggles with illness*,* I find joy and contentment in every day*,* thanks to the comfort that spirituality brings me.’ (M2)*.

Spirituality evolves over time, gradually becoming less materialistic and improving with personal and life experiences. It’s a dynamic journey encompassing past, present, and future. Interview findings suggest that as individuals age, their focus shifts from personal concerns to others, and from health and family harmony to finding fulfillment in service and contentment.

*‘Spirituality shapes our outlook on life*,* promoting mental balance and positivity. Helping others showcases our self-worth and leads to spiritual serenity*,* highlighting the essence of spirituality—a cycle of mutual support and reciprocity.’ (N3)*.

*‘Spirituality centers on mental purity; clearer minds lead to higher spiritual levels.’(N1)*.

*‘Even though I have retired*,* I strive to conserve resources for those in greater need and coordinate volunteering in our community with friends*,* bringing fulfillment and happiness.’(F14)*.

### Spiritual needs

Spiritual needs are triggered by stressful events that reach the threshold of the individual’s endurance. The spiritual needs of older adults are shown in Table 2.

**Table 2:** We identified spiritual needs that are key to maintaining endurance in the face of stressful events, some of which were Met and some not

1	Harmonious family relationship	Q1: ‘If our children could come back more often and spend more time with us” (F1). Q2: ‘As long as our child is healthy and happy, we will be happy.’ (F5). Q3: ‘I prefer to stay in my own home, but I have to go to a nursing home. My child has no time, so I can’t disturb my child’s life.’(F3). Q4: ‘As long as my children are healthy and have a good family, it’s worth it.’ (F10).
2	Satisfactory living environment	*Q5: ‘Life is quite satisfactory. I live in the city*,* which is more convenient; I go for leisure back to the countryside*,* the country life. I quite enjoy this comfortable life.’ (F1).* *Q6: ‘My community is very quiet*,* and the environment is very good. Neighbours get along very well with each other*,* and there has never been any bad thing…….’(F15).*
3	Closeness to nature	*Q7: ‘Just want to be close to nature*,* a feeling of exhilaration*,* yes*,* just that feeling*,* a state of relaxation*,* not thinking about anything*,* very comfortable.’(F1).* *Q8: ‘I really want to see the colour of the sea and listen to the sound of the waves. I feel comfortable when I think of that scene.’(F3).* *Q9: ‘I have always wanted to travel*,* but I spent a lot of money in hospital for disease. I also want to save some money for my children……’ (F6).*
4	Religious belief	*Q10: ‘I often go to church*,* I need God……’ (F10).* *Q11: ‘We all believe in Buddhism here*,* so do I*,* but not completely*,* just as spiritual sustenance.’(F1).* *Q12: ‘Sometimes I sit cross-legged and listen to some Buddhist songs on the internet. I feel good after listening to them.’ (F5).*
5	Friendly relationships	*Q13: ‘I can talk with my neighbours every day. I have a good relationship with them. I don’t feel lonely.“(F2).* *Q14:’A neighbour is better than a distant relative……’ (F5).* *Q15: ‘I am a believer. I think the most important relationship is with the Lord. I value my brothers and sisters in the church more.’ (F10).*
6	Sense of achievement	*Q16: ‘I used to be a teacher. I love this job very much…….Now students often come to visit me*,* which makes me especially happy.’ (F2).* *Q17: ‘Among the older adults I care for*,* they want to be recognized for their deeds and contributions*,* especially by their families.’(N1).*
7	Sense of belonging	*Q18: ‘I hope my children can come back often*,* and my home will be like ‘home’*,* with hope.’ (F3).* *Q19: ‘A kind of faith power*,* we have been carrying on the spirit of the previous revolutionary era. We all sing songs of revolutionary power during COVID-19*,* which is particularly powerful.’ (F14).*
8	Forgive and be forgiven	*Q20: ‘Sometimes I can’t forgive myself*,* which is the most painful thing in my life. I didn’t take good care of my parents. When I wanted to do it*,* my parents were gone.’ (F3).* *Q21: ‘There are benefactors*,* and there are villains*,* but at this age*,* all is forgiven.’ (F8).* *Q22: ‘People live like the Lord to forgive everything. People should always forgive others*,* which makes you comfortable. Matthew 6:14–15: ‘For if you forgive men when they sin against you*,* your heavenly Father will also forgive you.…….’ (F10).*
9	Fulfillment of life wishes	*Q23: ‘I always want to see the world*,* but my body limits me.’(F4).* *Q24: ‘I wish I had finished college” (F7). ‘* *Q25: ‘Not being able to send my daughter abroad is the biggest regret……’ (F8).* *Q26: ‘My biggest regret is giving away my little girl*,* who has not fully forgiven me.’(F9).*
10	Hope of being respected	*Q27: ‘The doctor had a very bad attitude*,* and said loudly to me*,* ‘I can only tell you this thing once*,* you want to remember to remember*,* forget if you can’t’. We forget things especially easy. The doctor does not show respect. His attitude is very bad.’ (F5).* *Q28: ’My wife and I are also worried about being abused in a nursing home.’(F15).*
11	Purpose and meaning in life	*Q29: ‘I didn’t have time to think about the meaning or purpose before. I just kept on for my family and children. Now it is meaningful to make myself useful for society.’ (F6).* *Q30: ‘There is no point in people repeating eating and drinking every day. Do something for society*,* people also for you*,* this is very meaningful.’ (M1).* *Q31: ‘We have experienced so much in our lives; it’s like a book. Going to the countryside*,* the Cultural Revolution……we all experienced that. Now we often look back on ourselves*,* and we really admire ourselves*,* and if we were to write a book*,* we would have a complete life. Our book will be written very richly.’(M2).* *Q32: ‘I don’t live for myself. I want to live for the Lord. I want to preach the Gospel so that everyone can enjoy the love of the Lord.’(F12).*
12	Sense of security	*Q33: ‘I want my wife and daughter to take care of me. I miss them too. They are here. I am at peace; I am not afraid.’ (F6).* *Q34: ‘I want my son and wife to take care of me so I can rest assured.’(F7).*
13	Love and be loved	*Q35: ‘I feels good when someone has concern for me*,* especially when I’m sick. I hope my wife and children care. It warms my heart to be cared for.’ (F6).* *Q36: ‘No one cares about me. I have a home*,* but it’s like no home……’(F13).* *Q37: ‘Being loved and remembered makes life meaningful.’ (F10).*
14	Connection of life to afterlife	*Q38: ‘In the face of death*,* I hope for a change or a paradise or an afterlife.I will ask a priest to pray for me.’ (F22).* *Q39: ‘In my decades of working experience*,* few people have really accepted death*,* not many really.’ (N2).*

#### Harmonious family relationships

Harmonious family relationships and caring and supportive family environments are important aspects of the spiritual health of older adults with chronic disease. Friendly interactions with family members can help older people with chronic disease find meaning and value in life. Elderly individuals’ expectations for harmonious family relationships are reflected in their need for an ample amount of contact with their children in Chinese traditional context. They aim not to impose economic, emotional, or spiritual difficulties on their children and hope that they can have a harmonious and happy family.The very expectation of harmonious family relationships highlights the current struggles that many elderly individuals with chronic diseases are facing (Q1-Q4).

#### Satisfactory living environment

When people retire, they often hope to experience a peaceful and calm living environment, where a balanced lifestyle, a serene community, and positive interpersonal relationships can contribute to a deep sense of fulfillment and happiness. This suggests that a satisfactory living environment may involve not only physical comforts but also the intangible aspects of social harmony and personal contentment (Q5-Q6).

#### Closeness to nature

Participants want to become close to nature through tourism and enjoy physical and mental relaxation in nature. However, due to practical factors, some older adults cannot achieve this goal due to their age, health, financial status and the COVID-19 pandemic (Q7-Q9).

#### Religious belief

In the face of unsolvable problems, some participants find spiritual support through religious belief. Here, religious belief may not necessarily refer to full conviction, but rather to practices such as attending religious services (Q10-Q12).

#### Friendly relationships

Positive relationships with neighbors can encourage elderly individuals to “leave the house,” engage in community activities, enhance their social interactions, and reduce feelings of loneliness. In this context, close neighbors may contribute more to older adults’ well-being than distant relatives (Q13-Q15).

#### Sense of achievement

A sense of achievement for older adults may stem from both recognition by others and their own sense of satisfaction (Q16-Q17). Many older adults seek recognition, particularly from family members, for their life’s journey and endeavors. This recognition, along with reflecting on a fulfilling life and achievements, can bring them genuine contentment and fulfillment.

#### Sense of belonging

A sense of belonging comes from one’s family, society and country. Especially during the COVID-19 pandemic, the protection provided by the country also makes individuals have a strong sense of national belonging. However, some seniors also express concern about where they will live in the future, even though nursing homes are available (Q18-Q19).

#### Forgive and be forgiven

Older adults may be more inclined than younger adults to choose forgiveness and seek forgiveness (Q20-Q22). This tendency is influenced by a combination of factors, including life experiences, emotional maturity, the adoption of broader perspectives shaped by time and existential reflection, as well as religious beliefs. However, it is important to recognize that not all older adults may exhibit this inclination, as individual experiences and reactions to forgiveness can vary significantly.

#### Fulfillment of life wishes

Fulfillment of life wishes focus on personal wishes and those related to one’s children (Q23-Q26). These wishes span a wide range of areas, such as the desire to travel, to pursue education for oneself or one’s child, and to provide support for one’s offspring. These expressions highlight the complex relationship between individual aspirations and the unavoidable limitations imposed by the realities of life.

#### Hope of being respected

Many older adults hope for respect from medical staff (Q27-Q28). While some seek respect and express concerns about dismissive doctors or the fear of abuse in nursing homes due to chronic illnesses, these experiences may not be universal. For those who do share these concerns, they highlight the importance of dignity, safety, and compassionate care throughout healthcare, particularly for individuals facing ongoing health challenges.

#### Purpose and meaning in life

Before retirement, participants’ sense of purpose and meaning is often centered around their children and family, particularly when facing life challenges. However, after retirement, their focus shifts towards personal purpose and meaning, which may include aspects such as religious beliefs, rich life experiences, and contributions to society (Q29-Q32).

#### Sense of security

In the context of chronic illness or hospitalization, older adults often find security in the care and trust provided by their family members. This companionship can play a significant role in fostering a sense of peace and well-being, particularly during challenging health crises (Q33-Q34).

#### Love and be loved

Older adults with chronic diseases often have a deep yearning for love and affection, especially from their family members and relatives. For this group, feeling loved is essential in providing meaning and hope in their lives (Q35-Q37).

#### Connection of life to the afterlife

Health caregivers believe that the satisfaction of spiritual needs is embodied in spiritual and psychological needs to some extent. The needs of older adults with chronic disease are related to and interwoven with psychological and emotional needs, especially when facing life threats; they often show anxiety, fear and other emotions and need to reevaluate the meaning of life, accept death education and consider their religious beliefs (Q38). Older adults often exhibit a paradoxical attitude toward death. While they acknowledge the inevitability of aging and mortality, they simultaneously harbor a deep fear of death (Q39).

There is an imbalance between spiritual needs, especially religious belief needs, and death needs. Faced with life threats, patients are more likely to accept religious belief needs and connect life belief needs with life, while it is rare and difficult for patients to truly accept death.

### Spiritual resources

Spiritual resources consists of two aspects: inner strength and external strength. Some participants have strong spirituality and do not need external support, and these participants can solve problems using their inner strength. Their spirituality may be stronger than that of spiritual caregivers. Therefore, some older adults express satisfaction with themselves, which can explain why they have no spiritual needs. The spiritual perception of individuals increases with age, which is related to individual experience. The more individuals experience, the more profound their perception of the meaning and value of life. Therefore, spiritual growth is also related to experience, which may explain why some non-elderly people are more spiritual. Inner strength refers to the ability to meet individual needs, mainly spiritual capacity. Some older adults need external resources such as family/relatives/friends, health caregivers or religious beliefs to meet their spiritual needs.

#### Inner strength

Inner strength refers to an individual’s ability to navigate difficult times and adapt to life’s challenges. It is seen as a resource that contributes to overall health and well-being. This strength can manifest in various ways, the ability to accept challenges beyond one’s control, and the capacity to focus on positive aspects of life despite adversity. Inner strength is not a fixed trait, but rather a dynamic potential that varies according to life experiences, personal values, and coping strategies. For older adults, it can help them address spiritual needs by fostering a sense of agency and meaning in the face of challenges.

*‘When I face problems*,* I simply work to resolve them*,* and when they prove insurmountable*,* I relinquish them to fate.’ (M2)*.

*‘Everything in life has its positive and negative aspects*,* and I choose to focus on the positive side.’(F14)*.

#### External support

When older adults with chronic disease are unable to fulfill their spiritual needs through inner resources alone, they often turn to external support to reinforce their strength. This support can come in various forms, such as connecting with family, friends, and caregivers; seeking companionship; and nurturing spiritual beliefs. While family and caregivers are typically categorized as external support, other forms of support, such as spiritual practices or nature, can be more complex to categorize, as they may be both external influences and sources of inner experience, depending on the individual’s perception.

For example, the support from a spiritual group, where individuals share common beliefs or values, can enhance spirituality and strengthen one’s sense of resilience. This external support, though derived from outside sources, plays a crucial role in nurturing inner strength.

*‘Chaplains often visit to pray for patients*,* who then pray together after they leave. Similarly*,* cancer survivors and AIDS patients form support groups where they share information and support each other in their fight against illness.’ (N2)*.

However, the realization of spiritual needs through external support may also have unintended negative consequences. In some cases, individuals may resort to extreme practices in an attempt to fulfill their spiritual needs, which can be harmful. When the wrong methods are employed, these actions can lead to suffering or other negative outcomes.

*‘It can be considered heretical; a person who leads a life of self-denial and extreme austerity*,* among other practices*,* may harm themselves and society as a whole.’(N3)*.

### Spiritual growth

Spiritual growth refers to the power and sense of accomplishment that an older adults with chronic disease experiences in an event or suffering through internal strength or external support.

*‘I wake up every morning at 4:30*,* and I begin my day with prayer. Each day*,* I experience a distinct sense of renewal and increased freshness.’(F12)*.

*‘Spirituality can evolve and develop*,* whether through religious beliefs*,* prayer*,* or self-training*,* all of which can contribute to enhancing my spirituality.’ (F23)*.

### Spiritual equilibrium

Spiritual equilibrium refers to the older adults with chronic disease using the spiritual growth acquired through life experience to overcome various difficulties to achieve excellence and blessings in life. Through hard work, ancestors have passed on admirable traditions, such as gratitude, dedication, altruism and other behaviours, through words and deeds. Those who have religious beliefs pass them on to others through preaching and other means. Those who have religious beliefs pass them on through faith.

*‘I am content with my life now*,* often feeling grateful*,* and I truly value my current life*,* thanks to the sacrifices of our ancestors. I’ll work hard to pass on this happiness to our descendants for an even better future.’ (M1)*.

*‘I always treat others as myself*,* helping those in need whenever possible. Over time*,* I’ve found joy in helping others*,* making it an integral part of my life.’ (F10)*.

### Spiritual outreach

Spiritual outreach consists of two aspects: deep outreach and broad outreach. Deep outreach refers to spiritual reflection (manifested more as life reflection). Broad outreach refers to the extension of spirituality, which is manifested in the individual’s willingness to help others, and the transfer of spiritual capacity to a group/event that needs the same, with the expectation that the group will also overcome all difficulties.

*‘Sharing your life experiences with others facing similar struggles brings more joy than overcoming your own challenges. In that moment*,* you realize every experience was worthwhile*,* as one person’s journey can inspire many.’(F13)*.

*The saying ‘I think about myself three times a day’ passed down from our ancestors is true: the more you focus on your own problems*,* the stronger your spirituality becomes.’ (F2)*.

Through repeated cycles, spiritual growth improves, thereby enhancing the inner strength of older adults with chronic diseases and strengthening their spiritual experience. The stronger an individual’s spiritual experience is, the more likely they are to comprehend spiritual awareness, thus forming a virtuous circle.

#### Spiritual cultivation

Older adults with chronic illnesses hope that hospitals could meet their spiritual needs. Health caregivers also indicate that spiritual practices should be strengthened, and they all hope to identify more ways to learn and cultivate spiritual knowledge in order to bridge the gap between the demand and supply of spiritual support. Participants who possess strong spirituality express a desire to further enhance their spirituality through education.

*‘I think it is necessary to provide spiritual care for patients. In clinical work*,* I have encountered many patients with terminal cancer pain suffering from illness. At this time*,* I should consider getting involved in spiritual practice….’ (N3)*.

*‘Spiritual training reduces fear*,* loneliness*, etc.,* and makes one’s limited life more meaningful. We should consider universal spiritual practice training.’(N1)*.

*‘Spirituality can be nurtured*,* leading to the development of greater inner strength. For instance*,* showing respect for the elderly and taking care of the young by fostering a culture of mutual assistance is essential. Neglecting this practicecan be quite perilous.’(M2)*.

## Discussion

Our findings contribute to the understanding of the spirituality of older adults. Six themes (spiritual awareness, spiritual needs, spiritual resources, spiritual growth, spiritual equilibrium, spiritual outreach and spiritual cultivation) promote the deep and comprehensive understanding and development of spiritual needs. The model of the spiritual experience of older adults provides guidance for the spiritual needs of older adults from theory to practice. It represents considerable progress for spiritual research.

We found that spirituality is one of the most basic aspects of humanity [31–32], which agrees closely with the definition of spirituality of the International Consensus Conference in 2014 [6]. Some older adults had relatively strong spirituality, which was in line with the spiritual development stage of the elderly population [33]. However, better spirituality does not mean that one has no spiritual needs, everyone has spiritual needs, especially in regard to major stressful events or ongoing traumatic events, such as crisis or illness [34–35]. Spirituality can help older people face all the hardships of life and embody the meaning and value of life, peace and contentment.

This study presents a novel dynamic spiritual development model that advances beyond traditional theories of post-traumatic growth [36] and gerotranscendence [37]. The framework makes three significant theoretical contributions: First, it identifies chronic illness in aging as a unique spiritual catalyst characterized by gradual, cyclical processes of existential meaning reconstruction and transcendence of suffering, distinct from the abrupt transformations following acute trauma. Second, it uncovers a culturally-contextualized reciprocity between individual resilience and community-based spiritual practices within Chinese sociocultural settings. Third, it delineates a developmental progression from implicit to explicit spiritual awareness. The model could provide an integrative framework for comprehending spiritual adaptation processes in diverse aging populations, while informing the design of culturally-grounded interventions that holistically address spiritual needs across the chronic illness continuum.

Spiritual needs vary based on individual uniqueness, so achieving inner peace, satisfaction, and a deeper understanding of life’s purpose requires customization to cultural and personal traits [10]. We found that older adults’ spiritual needs may evolve with stress events [34–35]. For instance, chronically ill patients may initially seek inner peace, faith, and hope. However, as the disease progresses, they may express a need for life meaning. This mirrors the continuous evolution of their spiritual needs in their life journey, consistent with the holistic care model [38]. However, older adults may face challenges in articulating their spiritual needs, partly due to limited spiritual sensitivity. Furthermore, opportunities for communication with healthcare professionals regarding spirituality are often scarce, as short hospital stays and frequent medical procedures exacerbate this difficulty, hindering patients’ ability to express and explore their spiritual needs during hospitalization.Therefore, advancing research on the spiritual needs of older adults should begin with establishing mechanisms and platforms for expressing their spiritual needs.

Spiritual growth is a continuous, evolving journey that forms an essential part of every individual’s life mission. Throughout the aging process, individuals are prompted to explore and engage with their spiritual needs and experiences to varying degrees, regardless of their awareness of spirituality. The spiritual journey is not linear, but rather spirals through cycles of growth, equilibrium, and outreach, as individuals adapt to their evolving life circumstances. None of the interviewees objected to the provision of spiritual care services; on the contrary, most expressed a strong desire for hospitals to address their spiritual needs. Healthcare providers acknowledged the importance of strengthening spiritual practices and emphasized the need to explore more avenues for learning and cultivating spiritual knowledge to better meet the growing demand for spiritual care.Participants with strong spirituality expressed a desire to enhance others’ spiritual well-being through education and support, further recognizing that spiritual growth is not a static experience but a dynamic process of continuous reflection and adaptation. This process allows individuals to navigate their shifting spiritual needs, fostering a sense of mutual benefit, inner peace, and shared growth. The spiral nature of spiritual development suggests that as individuals move through different phases of their life, they revisit and build upon previous stages of growth, always striving to align their spirituality with their values and needs.Spiritual outreach, while an essential component of spiritual growth, can bring both positive and negative effects. It is crucial that spirituality remains aligned with an individual’s core values and needs, and does not impose external beliefs onto others [39]. True spiritual growth should always promote well-being, fostering peace, understanding, and collective benefit. The dynamic, spiraling nature of this process enables individuals to grow and adapt in ways that enhance their own well-being and contribute positively to those around them.

A strength of the study is that it explores the understanding of spirituality from a secular perspective. Previous studies have focused on spirituality from religious perspectives, which could limit the transferability of the findings. Meanwhile, the study pays attention to the role of individual inner spiritual resources and their supplementation with external spiritual resources, which is conducive to achieving the balance of supply and demand based on the urgent spiritual needs in the context of increased ageing.

From an ethical perspective, we understand that addressing the spiritual needs of older adults with chronic conditions necessitates a nuanced approach grounded in medical ethics, with a particular emphasis on respecting multiculturalism and cultural sensitivity. Healthcare professionals are obligated to uphold the principles of respect, non-maleficence, beneficence, and justice. Respect entails acknowledging older adults’ cultural identities, beliefs, and values to prevent misunderstandings and foster effective spiritual support. Non-maleficence dictates avoiding actions that may harm older adults psychologically or emotionally, ensuring that interventions are safe, non-invasive, and culturally appropriate. Beneficence centers on promoting well-being through the fulfillment of spiritual needs, necessitating empathy and personalized support. Justice ensures equitable access to spiritual care, regardless of an individual’s background, status, or position. To accomplish this, professionals must continually enhance their cultural sensitivity and communication skills to improve culturally sensitive spiritual care for older adults.

This study underscores the complexity and individuality of spiritual growth, recognizing that it is not a one-size-fits-all process. Spiritual experiences can vary greatly, shaped by personal, cultural, and situational factors. Not all patients will experience spiritual growth; some may endure spiritual suffering without finding a path to transformation. Acknowledging this diversity is essential for understanding the range of spiritual journeys that patients may navigate.The goal is to foster an environment where spiritual suffering is acknowledged and addressed, and where patients are supported in a way that respects their unique cultural values and beliefs. While this approach is undoubtedly complex, particularly in multicultural settings, it is crucial for holistic care. Embracing the diversity of spiritual experiences and promoting spiritual well-being through culturally sensitive care is fundamental to patient-centered practices. This challenge should drive the continuous refinement of our care models, ensuring that each patient’s spiritual needs are met in a respectful, meaningful, and individualized manner.

### Implications

This study highlights the need to address the spiritual needs of older adults with chronic diseases in multicultural contexts, emphasizing the importance of culturally sensitive, tailored interventions for promoting spiritual well-being, with implications for practice, policy, and research.

Healthcare providers must integrate spiritual care into managing older adults with chronic diseases, recognizing spirituality as a dynamic aspect of aging. Interventions should consider cultural and spiritual backgrounds, promoting resilience and emotional well-being. Professionals need training to identify spiritual needs and ensure dignity and autonomy.

Cultural sensitivity is vital in multicultural contexts. Respecting diverse spiritual beliefs enhances intervention effectiveness. Healthcare systems must prioritize culturally competent care, and policies should promote training for providers to support the well-being of older adults from varied backgrounds.

The study calls for integrating spiritual care into national public health strategies for older adults. With chronic diseases prevalent, policies should ensure access to spiritual care, support resource development, provider training, and interdisciplinary collaboration.

This study opens new research avenues on spiritual experiences in older adults with chronic diseases. Future studies should examine the long-term impact of spiritual growth on health outcomes, and the role of spiritual care in resilience and coping. Research on culture, spirituality, and chronic illness will refine care models for diverse populations.Therefore, addressing the spiritual needs of older adults in multicultural settings is crucial for equity, respect, and dignity, enhancing quality of life and fostering a compassionate healthcare system.

### Limitations

We endeavored to encompass a diverse range of older adults with chronic diseases to ensure participant diversity. Additionally, this study contributes to filling the gap in spiritual research within secular society, bridging the divide between theoretical discourse and practical understanding. Consequently, the findings are both novel and significant. However, certain limitations must be acknowledged. Firstly, regarding sample representativeness, our efforts might have been insufficient in capturing the full spectrum of heterogeneity among older adults, including variations in cultural backgrounds, socioeconomic factors (such as income, occupation, education), disease severities, and urban-rural differences.Secondly, while incorporating semi-structured in-depth interviews and inductive content analysis has proven invaluable in developing our theoretical model, enabling deep insights into older adults’ complex experiences and perspectives, this methodology faces limitations rooted in subjectivity and interpretive bias during content analysis. To address these challenges and enhance the reliability and validity of our findings, we propose expanding the study population to include, for instance, older adults living alone, and refining our methods by implementing multiple coders or analytical triangulation. Furthermore, integrating diverse analytical approaches, including quantitative or mixed-methods research designs, can minimize individual biases and ensure a more balanced and comprehensive understanding of the data, thereby strengthening our research endeavors.

## Conclusion

Our research findings emphasize the significant spiritual needs of older adults, which are deeply rooted in traditional Chinese cultural values. Based on these insights, we’ve developed a theoretical model of spiritual experience tailored to this population, significantly advancing spirituality within nursing disciplines. To enhance spiritual health, we recommend raising awareness among healthcare professionals and older adults across diverse cultural backgrounds to accurately identify spiritual needs, implementing comprehensive training in spiritual research theories and practical applications to optimize resource allocation, and organizing social and spiritual activities that encourage participation and expression, thereby fostering spiritual growth and overall well-being.

## Electronic supplementary material

Below is the link to the electronic supplementary material.

Supplementary Material 1

## Data Availability

Data is provided within the manuscript or supplementary information files.
