# Supplementary material for: Exploring the spiritual experiences of older adults with chronic diseases: a qualitative study in a multicultural context
Source: BMC Psychol. 2025 Jul 1;13:632. doi: 10.1186/s40359-025-02710-3 (PMC12210451; doi:10.1186/s40359-025-02710-3)
Supplement: Supplementary file 1 — Supplementary Material 1 [file 40359_2025_2710_MOESM1_ESM.doc]

**Topic guide for older adults with chronic diseases and health caregivers**

| **Older adults with chronic diseases**  1. Have you ever heard of spirituality? How do you understand spirituality? Can you talk about it?  2. When do you think deeply, or what events do you think deeply about? Do you think these events are spiritual? If so, why? If not, why not?  3. How do you evaluate the current state of your life?  4. What keeps you going in the face of a crisis? Can you talk about it?  5. How do you consider the purpose and meaning of life? When or what makes your life meaningful?  6. When do you feel most at peace with yourself?  7. How do you view relationships?  8. So far, what do you think you want to do most?  9. How do you feel about religious belief? When do you think about religion?  10. Do you think spiritual care should be provided in healthcare? If so, why? If not, why not?  11. How do you feel about spiritual care? What spiritual care would you like?  Based on their answers, we will ask in-depth questions  **Health caregivers**  1. Have you ever heard of spirituality? How do you understand it?  2. How do you focus on the spiritual needs of your patients?  4. What do you think are the spiritual needs of patients?  5. Have you explored how patients cope with spirituality?  6. Do you feel the need to provide spiritual care to patients? If so, why? If not, why not?  Based on their answers, we will ask in-depth questions |
| --- |
